# Artificial inteligence and datasets for leukemia diagnosis: A scoping review of machine lerning and deep learning approaches

**DOI:** 10.1016/j.mex.2025.103722

**Published:** 2025-11-13

**Authors:** Ashwini Tande, Renuka Mane

**Affiliations:** Department of Computer Engineering and Technology, Dr. Vishwanath Karad MIT World Peace University, Pune, India

**Keywords:** Leukemia diagnosis, Deep learning, Peripheral blood smear, WHO 2022 Classification

## Abstract

Leukemia is the cancerous disease of the blood and the bone marrow that causes excessive proliferation of abnormal white blood cells, if detected too late, it can lead to potentially fatal consequences. Peripheral blood smear examinations and bone marrow biopsy are example of conventional diagnostic techniques that are invasive, time-consuming and subject to human variability. Recent advances in artificial intelligence (AI) particularly in the areas of Machine Learning (ML) and Deep Learning (DL)offer encouraging answers by making it possible to detect and classify leukemia using automated, effective and precise techniques.

With an emphasis on image-based techniques based on publicly available datasets such as ALL-IDB, C—NMC, AML_Cytomorphology_LMU, SN-AM and CPTAC-AML, this paper reviews the most recent research on Machine Learning and Deep Learning approaches includes Convolutional Neural Networks (CNNS), ResNet, DenseNet, MobileNet and ensemble models for leukemia diagnosis. The survey highlights some of the most significant issues, such as dataset imbalance, stain variability, lack of standard annotations and limited clinical validation. The paper also discusses research gap and future initiatives including Explainable AI, lightweight deployment models, clinically reliable diagnostic system and hierarchical classification framework aligned with WHO 2022 classification standards.

## Highlights

• Detailed domain knowledge required to understand blood formation process, leukemia, WHO classification standards and standard for diagnosis.
• Detailed Analysis of publicly available benchmark datasets for leukemia diagnosis.
• Review of ML and DL studies for leukemia detection and classification.
• Research gaps including dataset imbalance, WHO 2022 classification alignment.

## Specifications table

**Table:** 

Subject area	Computer Science
More specific subject area	Medical Imaging
Name of the reviewed methodology	Scoping Review
Keywords	Leukemia Diagnosis; Deep Learning; Peripheral Blood Smear; WHO 2022 Classification;
Resource availability	Elsevier, IEEE Access, Wiley
Review question	RQ-1: What fundamental domain knowledge is required to develop CAD for Leukemia? RQ-2: What is the international standard for diagnosis of leukemia? RQ-3: What are the frequently utilized and publicly available datasets? RQ-4: Which CNN models and other Machine Learning techniques are used for feature extraction and classification? Which performance metrics are used? RQ-5: What are the challenges and gaps are there in existing system?

## Background

Cancer is a group of diseases caused by the abnormal growth of cells which can initiate in nearly every organ or body tissue when uncontrolled growth of cells takes place [1]. Globally, cancer ranks as the second leading cause of death. Every year, millions of new cases of cancer are occurring which is increasing the disease’s burden worldwide. Cancer has tremendous impact on society and the economy. Patients frequently have lower life expectancy and quality of life, which turns worse by the high expense of late-stage treatments. These difficulties highlight the necessity of precise and timely detection [2].

As per Globocon 2022 reports, over 19.98 million cases with cancer were diagnosed and recorded recently, resulting in over 9.74 million deaths worldwide [3]. 1.41 million incidents and 0.91 million deaths due to cancer were recorded in India [5]. Oral cavity, lung and stomach cancer are common in male, Breast, ovary and cervix uteri cancers are common in female [4] and children are mostly affected due to Leukemia, Lymphoma and brain and CNS cancers. Leukemia alone accounts for 2.5 %of total cancer incidences and 3.1 % of total cancer-related deaths globally [6]. The worldwide leukemia incidence is 487,294 and the deaths are 305,405 as per Globocon 2022 report. In India 49,883 are new cases and 36,871 deaths due to leukemia [7].

Hematologic malignancies in oncology include leukemia, lymphoma and multiple myeloma are blood cancers that arise from the malignant transformation of a single cell in the spleen, lymph nodes or bone marrow [8]. The lymphatic system, which eliminates extra fluid and generates immune cells, is the site of lymphomas, which account for half of all blood malignancies each year. As abnormal lymphocytes develop into lymphoma cells, the immune system gradually deteriorates[9]. Myeloma is a type of cancer that affects plasma cells which are responsible for producing anti-infective antibodies. The immune system is weakened by this overproduction, which also interferes with RBCs and WBCs function. Often called multiple myeloma, myeloma cells can also spread through the blood and gather in other bones [10].

Early and precise diagnosis is essential as delayed diagnosis lowers survival rates, raises treatment complications and worsens prognoses. Morphological analysis, cytogenetics and immunophenotyping are examples of traditional diagnostic techniques that are time-consuming, arbitrary and reliant on professional interpretation, this leads to a high demand for scalable, automated and reproducible solutions.

Collaboration between academics, medical practitioners and policymakers is crucial for Artificial Intelligence (AI), Machine Learning (ML), Deep Learning (DL) [11]. ML and DL are revolutionizing leukemia diagnosis by addressing the limitations of traditional methods. These technologies offer automation, accuracy, efficiency and accessibility.

### Method details

This review aims to the readers who are at initial phase of the research journey in diagnosis of hematology in oncology using AI. The articles search is done based on keywords “Acute Lymphoblastic Leukemia” OR “Acute Myeloid Leukemia” AND “classification” OR “detection”, AND “subtype classification” AND “Deep Learning” for CAD of leukemia. For the fundamental domain knowledge of leukemia, the keywords used are “White Blood Cells Morphology”, “Leukemia classification”, “WHO”, “International Standard for Diagnosis”.

The research articles based on detection and classification are included since 2020. The articles before 2019 are excluded. For the domain knowledge of leukemia articles are considered since 2016 as in 2016 WHO has revised the diagnosis standard and subtype classification criteria, and latest it is revised in 2022. Relevant web articles are also considered for basics of blood. The articles that only include other hematologic malignancies such as myeloma, anemia, lymphoma is excluded from the study. The focus of this article is mainly on Leukemia and it’s subtypes.

The following research questions made this review clear and structured:

1. RQ-1: What fundamental domain knowledge is required to develop CAD for Leukemia?
2. RQ-2: What is the international standard for diagnosis of leukemia?
3. RQ-3: Which are the frequently utilized and publicly available datasets?
4. RQ-4: Which CNN models and other Machine Learning techniques are used for feature extraction and classification? Which performance metrics are used?
5. RQ-5: What are the challenges and gaps are there in existing system?
6. RQ-6: Is the international standards for classification and diagnosis are considered?

## Leukemia overview

### Blood formation process and the leukemia

Before going into details of WBCs and its malignancies we need to know how blood is produced. The bone marrow produces stem cells and blood formation begins. These stem cells differentiated into two cell lines: lymphoid and myeloid. In the lymphoid cell line develops B cells, T cells, Natural Killer (NK) cells and plasmacytoid dendrite cells are produced which all plays important role into immune system and are classified as agranulocytes and a part of WBCs. Alternatively, when stem cell takes the myeloid line, it differentiates into a greater number of cell types. They give rise to RBCs (erythrocytes) that carries oxygen, platelets (thrombocytes) which aid in clotting blood and myeloblasts that matures into granulocytes a part of WBCs which are categorized as neutrophils, eosinophils and basophils. Thus, platelets and RBCs develop only from myeloid lineage whereas WBCs develop from both myeloid and lymphoid lineage[12,13].

**Fig. 1:**
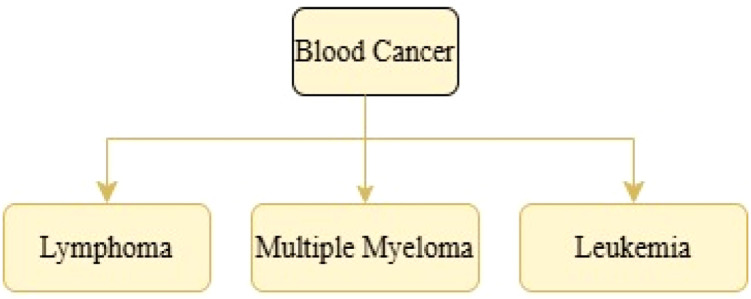
Blood Cancer Taxonomy.

Stem cell is responsible for the formation of RBCs (erythrocytes)s, WBCs (leukocytes) and platelets (thrombocytes) in bone marrow. The bone marrow, where stem cells develop into RBCs, WBC, or platelets, is usually where blood malignancies start. Fig. 1 shows common hematologic malignancies. The lymphatic system is impacted by lymphoma, a blood cancer that eventually weakens the immune system by causing aberrant cells. The malignancy known as myeloma impairs the body’s ability to produce antibodies normally, which weakens the immune system and leaves it vulnerable to infection. This paper focuses on the type of blood cancer that has rapid generation of aberrant white blood cells known as leukemia, a type of cancer that can be found in the bone marrow and the blood. The bone marrow’s capacity to create red blood cells and platelets is hampered by the large number of aberrant white blood cells [14].

Leukemia occurs when a stem cell undergoes a leukemic change due to mutation. It causes abnormal cells to grow and survive better than normal cells which supress the development of healthy blood cells in the bone marrow [15].

### White blood cell structure

Leukocytes (WBCs) are classified as granulocytes and agranulocytes. This classification is done based on the presence and absence of microscopic granules in their cytoplasm.

Granulocytes are classified mainly into Neutrophils, Eosinophils, Basophils. It’s precursor and immature forms are Myeloblast, promyelocyte, myelocyte and metamyelocyte. These granulocytes are differentiated from one another by their nucleus’s morphology, size how their granual is stained. Polymorphonuclear neutrophils, another name for neutrophils, with multilobed nuclei with three to five segments connected by thin strands and measures 12 to 15 µm in diameter. Their cytoplasm is a pale pink tint due to certain granules that are not visible under light microscopy. Large cytoplasmic granules that are eosinophilic and stain red to pink are found in the biolobed nucleus of eosinophils. Basophils feature

An S-shaped or bilobed nucleus, measures 12 to 15 µm in diameter and contain cytoplasmic specialized granules that stain blue to purple[16].

Agranulocytes are divided into lymphocytes and monocytes. Its precursor variants are lymphoblast, Atypical lymphocyte, monoblast, promonocyte and smudge cell. Monocytes which make up 4–8 % of WBCs, are progenitor cells for the mononuclear phagocytic system. They feature huge indentation or C—C shaped nuclei, measure 12 to 15 µm in diameter that has a lot of cytoplasm that is bluish-grey in hue because of lysosomal granules at light microscope resolution. 25 % of WBCs are lymphocytes which are round and appear in different sizes. Like RBC, small lymphocytes contain spherical, heterochromatic nuclei. Like active cells, larger lymphocytes are 9–18 µm in diameter and feature indentation nuclei. CD markers are used to separate them into B and T cells [16].

### Classification of leukemia

Leukemia is classified in two ways:

Based on cellular line of origin as Myeloid (also termed as myelogenous) and Lymphoid (also termed as lymphocytic or lymphoblastic).

Based on the rate of proliferation of malignant cells as Acute and Chronic[17].

When the leukemia develops in the lymphocytes (lymphoid cells), it is called lymphocytic leukemia and when it develops in granulocytes or monocytes (myeloid cell), it is called myeloid/ myelogenous leukemia.

So, based on above factors Leukemia can be broadly divided into four categories:

1. Acute lymphoblastic/lymphoid leukemia (ALL)
2. Acute myeloid/myelogenous leukemia (AML)
3. Chronic lymphoblastic leukemia (CLL)
4. Chronic myeloid leukemia (CML) [18]

There are several revisions made on the classification system of Leukemia. WHO revises classification system for hematolymphoid tumors based on certain things. The very first classification is FAB (French-American-British) classification system which is published in 1976. WHO in association with Society of Hematopathology and European Association of Hematopathology has published new classification system for hematopoietic and lymphoid neoplasm in 2001 A basic principle of the WHO system is that the classification of hematopoietic and lymphoid neoplasms should utilize not only morphologic findings but also all available information, including genetic, immunophenotypic, biologic, and clinical features to define specific disease entities[19]. Later in 2008 WHO has revised the classification system and new entities were added in 4th edition like molecular genetics in diagnosis. Also therapy related neoplasms were recognized as distinct group [20]. In 2016 revision to 4th edition is published where new genomic advancements are advised such as (CALR, SF3B1, CSF3R, TP53, NPM1, CEBPA), lineage based and morphology focused classification improving diagnostic accuracy [21]. Recently in 2022 WHO has shifted the classification system from morphology driven towards genetic, molecular and biologically informed classification. AML classification is now clearly separated as AML with genetic abnormalities and AML by differentiation [[22], [23], [24]]. Table 1 shows the Leukemia classification and sub-type classification as per the mutations and WHO 2022 classification standards for Leukemia.

**Table 1:** Leukemia classification system as per WHO 2022 standards.

Category	Subtype	Representative Features / Subclassifications
**1. Acute Myeloid Leukemia (AML)**	a. AML with defining genetic abnormalities	Includes fusions such as *RUNX1::RUNX1T1, CBFB::MYH11, KMT2A, NUP98, NPM1*, and others.
	b. AML defined by differentiation	Based on morphology and immunophenotype when no defining mutation is found
	c. AML, myelodysplasia-related	Previously AML-MRC; often evolves from MDS or shows MDS-type mutations
	d. Therapy-related AML	Arises after chemotherapy/radiation; usually high-risk
	e. AML with germline predisposition	Includes *TP53, CEBPA, SAMD9/SAMD9L*, Down syndrome-related AML
**2. Acute Lymphoblastic Leukemia (ALL)**	a. B-lymphoblastic leukemia (B-ALL)	Subtypes include *ETV6::RUNX1, BCR::ABL1, KMT2A, TCF3::PBX1*, and others based on molecular features
	b. T-lymphoblastic leukemia (T-ALL)	Includes Early T-cell precursor ALL; lacks the diversity of B-ALL in current molecular subclassification

As per French-American-British classification ALL is divided into L1, L2 and L3. Small and uniform, L1 blasts have spherical, regular nuclei and subtle nuclei. Large and diverse, L2 blasts have a lot of cytoplasm and uneven frequently clefted nuclei. Regular, round-oval nuclei and conspious nucleoli characterize L3 blasts, which are moderately big and uniform [25]. WHO categorises ALL into two subtypes: B-cell ALL and T-cell ALL. B-cell ALL is further divided into Early pre-B ALL, pre-B ALL and pro-B ALL[26].

The FAB classification system categorizes AML into eight subtypes [27].

1. M0 Minimally differentiated AML
2. M1 AML with minimal maturation
3. M2 AML with maturation
4. M4 Acute myelomonocytic leukemia (AMML)
5. M5 Acute monocytic leukemia (AMoL)
6. M6 Acute Erythroid Leukemia (Erythroleukemia)
7. M7 Acute megakaryoblastic leukemia (AMkL)

**Fig. 2:**
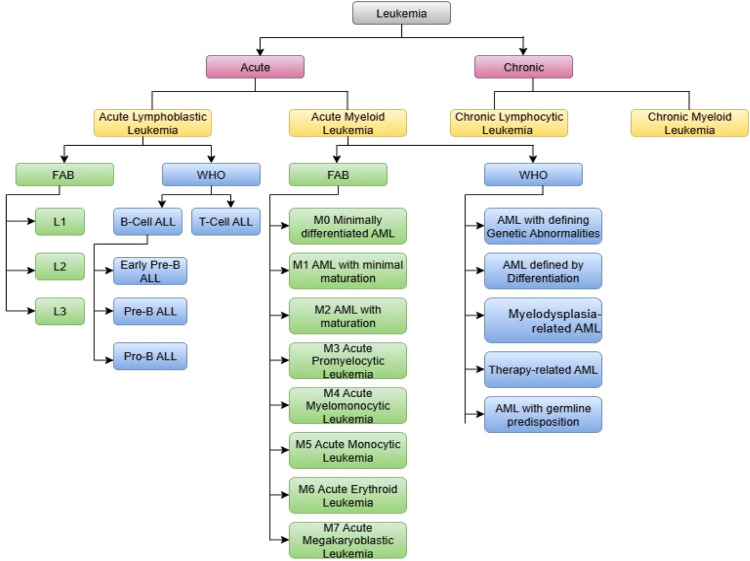
Taxonomy of Leukemia.

As per WHO 2022 classification system AML is classified into five groups [23]:

1. AML with defining genetic abnormalities
2. AML defined by differentiation
3. Myelodysplasia-related AML
4. Therapy-related AML
5. AML with germline predisposition
6. As per revised WHO classification system 2022, leukemia classification is shown in table and detailed FAB and WHO classification system is shown in Fig. 2.

### Causes, symptoms and international standard for diagnosis of leukemia

Leukemia has various genetic, environmental, and lifestyle risk factors, though its exact causes remain unknown in many cases. Acute Myeloid Leukemia (AML) is linked to mutations such as FLT3-ITD, NPM1, and TP53, as well as prior chemotherapy, radiation, and benzene exposure [28,29]. Acute Lymphoblastic Leukemia (ALL) is associated with mutations like IKZF1 and PAX5, along with radiation and pesticides, but its exact causes remain unclear in many cases [30]. Chronic Lymphocytic Leukemia (CLL) has a strong genetic component, though no single definitive cause has been identified [31]. Chronic Myeloid Leukemia (CML) is driven by the BCR-ABL1 fusion gene but why this mutation occurs is still uncertain [32].

Symptoms generally include fatigue, infections, bleeding, fever, night sweats, and weight loss. AML often presents bone pain and organ enlargement, while ALL commonly involve swollen lymph nodes and fever, particularly in children. CLL symptoms include prolonged fatigue, weight loss, and frequent infections. CML is characterized by fatigue, night sweats, and spleen enlargement, often detected in its chronic phase [33,34].

The diagnostic process for acute leukemia begins with Complete Blood Count (CBC) and Peripheral Blood Smear (PBS). If suspected, a Bone Marrow (BM) evaluation is performed with aspiration or biopsy. Bone marrow aspiration is the process where fluid is collected from patient’s body whereas in bone marrow biopsy small tissue sample of bone marrow is collected which is quite painful. Morphological assessment is done with PBS and BM aspirations and biopsy reports [35,36]. After the diagnosis of acute leukemia, the immunophenotyping via flow cytometry is performed to check the lineage and to detect the sub-type of the leukemia [37,38]. With each type of leukemia patients, the treatments undergo differently. So, it is essential to diagnose the leukemia and type and sub-type of leukemia at early stage, which is possible with advances in technologies by reading PBS images after completing CBC. Advances in AI technologies enable early diagnosis, type and subtype classification using the PBS and BM images [39].

Although ALL can affect both children and adults, it is most common in children aged two to five. AML is mostly occurred in adults with median age 68 at the time of diagnosis[35,40].

## Literature for datasets and ML-DL models

### Datasets

Generally, an automatic detection can have several steps such as segmentation, identification of morphological attributes of WBCs, identification of lymphocytes etc. There are several datasets available which mentioned in Table 2 and explained below.

**Table 2:** Publicly available Leukemia Datasets.

Ref No	Dataset Name	Modality	No. of Samples and pixel size	Label Types	Imbalance type
[25]	ALL-IDB1	PBS Images	108 images with 39,000 blood elements with size 2592×1944 with 510 candidate lymphocytes	L1, L2, L3 B-ALL	Balanced
	ALL-IDB2	PBS Image (Microscopic)	260 images with 260 blood elements with size 257×257 and 130 candidate lymphocytes		Balanced
[42]	C-NMC Dataset	Bone Marrow aspirates images (Microscopic)	15,114 images from 118 subjects	B-ALL	Imbalanced (49 healthy, 69 cancer)
[43]	SN-AM	Bone Marrow aspirates images (Microscopic)	90 B-ALL, 100 MM	B-ALL, Multiple Myeloma	Blaanced
[44]	Acute Lymphoblastic Leukemia Image Dataset	PBS images	3256 with 89 ALL subjects and 25 healthy subjects	Early pre-B, pre-B, pro-B	Imbalanced (higher number of ALL cases than benign)
[45]	AML-cytomorphology_LMU	Single cell PBS images	18,365 single-cell PBS images form 100 patients and 100 healthy individuals	15 Morphological classes with abbreviations	Balanced
[46]	CPTAC-AML	Bone marrow	180 subjects, 88 AML patients, 50 bone marroe smear with M1(31 cases), M2 (19 cases)	M1, M2	Imbalanced

#### ALL IDB1 and ALL IDB 2

ALL-IDB is a publicly available dataset made available for developing and evaluating image processing algorithms for leukemia detection, particularly segmentation and classification of white blood cells. This dataset was presented and published in IEEE international conference on Image Processing held at Brussels, Belgium in 2011. It has two subsets: ALL-IDB1 and ALL-IDB2. ALL-IDB1 contains 109 high-resolution images (2592×1944 pixels) of peripheral blood samples, with 39,000 blood elements and including 510 labeled lymphoblasts, used for testing segmentation and classification algorithms. ALL-IDB 2 is composed of 260 cropped images (257×257 pixels) with 50 % containing lymphoblasts, making it more suitable for classification task. The dataset also incorporates morphological features critical for distinguishing normal lymphocytes from lymphoblast, following the FAB classification: L1, L2, L3. It also highlights general morphological traits, such as nuclear shape, cytoplasmic content, and irregular cell structures [25].

#### C—NMC

The B-ALL blood cancer dataset, prepared at the Laboratory Oncology unit at AIIMS, New Delhi, India. It contains 15,114 cell images of 2560×1920 pixel in the BMP format from 118 subjects. The slides were prepared from the subject’s Bone Marrow Aspirate. The images were captured using NIKON microscope mounted with NIKON DS5M camera. These images are stain color normalized with GCTI-SN method and segmented using hybrid segmentation technique that combines K-means clustering in Lab color space with ridge detection via 4-layer deep belief network (DBN) which segments overlapping WBC nuclei [41]. The dataset is divided into training and testing sets, with training set containing 12,528 images from 8491 lymphoblasts and 4037 healthy blasts, and the test containing 2586 images from 8 healthy and 9 cancer patients. The dataset is released during the IEEE ISBI 2019 medical imaging challenge and also released at the TCIA following standard protocols by TCIA. This is the largest cell imaging dataset in the public domain for B-ALL cancer classification problem.

However, the dataset faces challenges such as dataset imbalance, as the cancer samples are more than double the healthy class. Despite medical experts preparing the ground truth or annotating the dataset, there is still possibility of label noise in cancer class [42].

#### SN-AM

This dataset involves microscopic images collected from bone marrow aspirate slides of patients with B-ALL and Multiple Myeloma (MM) obtained from clinical pathology services. The pictures were taken at 1000x magnification with a Nikon Eclipse-200 microscope in raw BMP format. There are 30 images in the collection, 29 of them were examined using the stain normalization technique and one image served as the reference image. The nucleus and background masks are included in two extra photos for every image. For instance, “ALL_1_nucleus _mask.bmp” is the image with the mask on the nuclei and “MM_1_background_mask.bmp” is the image with mask on the background if the original file is saved with the name “ALL_1.bmp”, respectively. The dataset is divided into two subsets where dataset subset-1 contains 90 images from cases of B-ALL and dataset subset-2 contains 100 images from cases of MM which are saved in BMP format with a size of 2560×1920 pixels. The dataset is available on TCIA cancer repository [43].

#### Acute lymphoblastic leukemia (ALL) image dataset

This dataset is prepared at Taleqani Hospital’s (Iran) Bone Marrow Lab. The 3256 PBS images from this dataset were from 89 suspected ALL patients whose blood samples were processed and stained by knowledgeable lab personnel. The dataset is primarily divided into two classes: Benign and Malignant. Early pre-B, pre-B and pro-B ALL are three subtypes under malignant category. A professional identified the types and subtypes of these cells conclusively using the flow cytometry technology. Following segmentation in the HSV color space using color thresholding, this dataset offers segmented images. The images are of size 1024×768 size saved in JPG format. Benign images are 504 and malignant images are categorized as Early Pre-B ALL 985, pre-B ALL 963 and pro-B ALL 804 images resulting in a total of 3256 images collected from 89 patients [44].

#### AML-Cytomorphology_LMU | A single-cell morphological dataset of leukocytes from AML patients and non-malignant controls

The Munich AML morphology dataset comprised of 18,365 single-cell images of peripheral blood smears from 100 patients diagnosed with Acute Myeloid Leukemia and 100 healthy individuals, at Munich University Hospital from 2014–2017, all labeled by experts. Images were obtained at 100-fold optical resolution and with oil emersion using the M8 digital microscope/scanner. Proposal of pathological and non-pathological leukocytes were done using the same morphological classification scheme that was used in clinical practice. To quantify inter and intra-rater variability, a subset of images was re-annotated up to two times. Each annotated cell image in the dataset is labeled with a brief abbreviation that corresponds to its morphological classes with two categories mature and immature leukocytes and further categorized as NGB (neutrophil band), NGS (neutrophil segmented), LYA (atypical lymphocyte), LYT (typical lymphocyte), BAS (basophil), EOS (eosinophil) as mature and EBO (erythroblast), KSC (smudge cell), MMZ (metamyelocyte), MOB (monoblast), MYB (myeloblast), MYO (myelocyte), PMB (bilobed promyelocyte), and PMO (promyelocyte) as immature leukocytes [45].

#### CPTAC-AML dataset

It contains bone marrow or peripheral blood smear images from 88 AML patients (122 tissue slide images) collected by the American National Cancer Institute. The classification of AML is based on FAB classification criteria [8]. A total of 50 bone marrow smear images from patients with M1 (31 cases) or M2 (19 cases) subtypes of AML were included in this study, and all enrolled patients had clinical data. The research was carried out in accordance with the World Medical Association Declaration of Helsinki and was approved by the Ethics Committee at The First Affiliated Hospital, and College of Clinical Medicine of Henan University of Science and Technology. The dataset includes abbreviations for morphological classes and and how the image dirctories are organized [46].

### Machine learning and deep learning approaches for leukemia diagnosis

[47] has developed a cluster-layer normalized CNN, ALNett model, that is suggested for ALL image classification from the ISBI 2019 dataset. Structural and contextual features are captured by the model with 99.73 % training and 91.13 % test accuracy.

In [48], it tackles ALL detection from microscopic images under dataset imbalance with C—NMC (highly imbalanced) and ALL-IDB2 (moderately imbalanced). Three approaches—input-based, GAN-based, and loss-based—are evaluated across VGG16, ResNet50, DenseNet121, and EfficientNetB0. Loss-based solutions excel in high-imbalance scenarios.

Based on the C—NMC_Leukemia dataset, a novel deep learning model named PLCT is introduced in [49], including preprocessing, feature extraction by AlexNet, and classification by an optimized CNN. The proposed model attains 99.99 % accuracy.

A Dilated Deep Residual Neural Network (DDRNet) is designed by [50] based on a public Kaggle blood image dataset. It comprises Deep Residual Dilated Block (DRDB), Global and Local Feature Enhancement Block (GLFEB), and Channel and Spatial Attention Block (CSAB), achieving 99.86 % training accuracy, 91.98 % test accuracy, and F1-score of 0.96.

[51] presents automated method to detect ALL, where AlexNet as a feature extractor and SVM as a classifier outperformed. MATLAB is used for implementation which quite uncommon approach in term of deployment that Python.

DL framework has been developed in [52], to classify ALL cell from normal WBCs using C—NMC dataset where five pre-trained CNNs are used for spatial features, GRU-BiLSTM for temporal features and MSVM as a classifier.

[53] has employed four benchmark datasets to classify ALL and AML by applying ResNet18 for feature extraction and an Orthogonal Softmax Layer (OSL) for classification, in place of the traditional fully connected layer. Accuracy figures dataset-wise are given in tabular format.

Fifty-six images from the blood smears in the ASH dataset are hand-cropped into 168 B-cell and 168 T-cell lymphoblast images in [54]. Augmented data are employed for training AlexNet and LeukNet, both of which obtain 94.12% accuracy. LeukNet is more efficient, taking 2–3 min versus 7–8 min by AlexNet. The method skips segmentation to go directly to classification.

Study in [55] aims to develop and validate DL model capable of distinguishing between AA, MDS and AML using bone marrow smear images. The model uses ImageNet-pretrained ResNet-50 CNN and has dual output structure for binary and ternary classification.

[56] developed MobileNetV2, a lightweight CNN that is used for feature extraction and classification is done using SVM with an RBF kernel that achieves best accuracy for ALLIDB1, ALLIDB2 and ASH datasets for binary classification.

[57] uses EfficientNetB7 and MobileNetV3Large with feature fusion-based stacked ensembling. The two-stage architecture involves feature extraction, feature fusion, image enhancement, transfer learning with frozen weights dataset fusion, interpretability with Grad-CAM that offers lightweight deployment potential. The approach also handles class imbalance through augmentation and careful dataset split.

[58] aimed to evaluate different CNN architectures with combination of different activation functions to classify subtypes of AML using the ASH dataset. The architectures incorporated are VGG16, InceptionV3 and ResNet50v2 where the best accuracy was achieved with InceptionV3 with GELU at 94.02 %, while ResNet50v2 was computationally efficient offering shorter training time with accuracy of 92 %. The activation functions tried were GELU, Mish, Softplus and Softsign.

In [59] k-means clustering is used with Enhanced Grey Wolf Optimization (EGWO) for the selection of features in a dataset of 3189 images of ALL subtypes. Out of all the models, SVM with EGWO has an accuracy of 99.22 %.

[44] presents an CNN-based classification to classify B-ALL subtypes, which extracts the features using DenseNet-201 after applying segmentation. The model is trained using two fully connected layers with NN on ALL image dataset available on Kaggle.

[60] developed a fine-tuned ResNet-50 model for ALL image dataset, available on Kaggle, enhanced with additional dense and dropout layers, employing data augmentation and five-fold cross-validation to improve generalization and avoid overfitting that achieved 99.38 % accuracy and f1-score, outperforming other pretrained CNNs like VGG-16, DenseNet-121 and EfficientNetB0.

[61] obtained 43 morphological, 276 radiomic, and 1 clinical feature from 50 bone marrow smear images in the CPTAC-AML (TCIA) dataset. A Random Forest model with nine features had a training AUC of 0.998 ± 0.004 and accuracy of 0.998 ± 0.003. The BLS model also had 0.973 accuracy. Though 0.8 accuracy on weakly stained slides is achieved, shortcomings are small dataset size, M1/M2 class imbalance, single clinical feature dependence, and lack of external validation.

[62] implemented a two-stage leukocyte classification model known as LeuFeatx uses an optimized VGG-16 feature extractor trained on the AML morphological dataset of 15 leukocyte subtypes. It is tested on the PBS-HCB dataset of Hospital Clinic of Barcelona with 8 cell types and the ALL-IDB2 dataset for generalization. XGBoost performs the best on the AML dataset with macro-average recall of 0.643 and F1-score of 0.605. For PBS-HCB, the model is accurate to 92 %, whereas on ALL-IDB2, the Extra Trees classifier is 96.15 % accurate.

[63] explores the number of images needed to train a CNN to accurately detect malignant leukocytes from single cell blood smear images and concludes that more data is not always better. The study used two datasets: ALL-IDB and AML-cytomporphology_LMU (available from TCIA). It performs binary classification on ALL and AML and evaluate the performance using ROC_AUC which highlights 30 images are sufficient for training with proper annotations as ROC_AUC was slightly improving with 50 images. It further performs multiclass classification for ALL morphological feature: Lymphoblast, Typical Lymphocyte, Atypical Lymphocyte, Neutrophil, Monocyte, Thrombocyte.

[64] first detects AML as immature blood cell and then classifies immature blood cell into Myeloblasts, Monoblasts, Erythroblasts, Promyelocytes. The framework combines Convolutional Autoencoders (CAE) for feature embedding and synthetic image generation, VGG19 and ResNet50 for robust feature extraction and weighted ensemble to enhance classification performance. The model outperformed in binary as well as multiclass classification.

[65] presents a novel hybrid method for improving WBC detection and feature extraction from microscopic images for AML diagnosis. The study used the primary dataset of 18,365 labelled single-cell images from Munich University Hospital and secondary dataset of 17,092 peripheral blood cell images. The proposed method involves CMYK-Moment Localization to cover RGB images to CMYK color space and localize WBC nucleus. CNN-based feature fusion method to produce 128-dimensional feature vectors per pixel. Four classifiers were used: FCL, RF, SVM and XGBoost, where CMYK + CNN feature extraction method outperformed with RF in primary dataset with 97.57 % accuracy and with SVM in secondary dataset with 96.41 % accuracy.

A mobile CNN application is implemented in [66] for B-ALL and its subtype identification from a Kaggle database of 3242 images of 89 patients. Thresholding and K-means clustering separate blast cells, and masking preserves the ROI. MobileNetV2 provides 100 % accuracy, precision, recall, and specificity.

For training, 1033 image bone marrow datasets (306 ALL, 500 AML, 162 CML, 294 lymphoma, 208 MDS/MPN, 272 MM, and 291 healthy) is employed in [67]. Model performance is analyzed on ALL-IDB1 and SN-AM datasets (Table 3).

**Table 3:** ML and DL techniques used for leukemia detection and classification.

Ref. No	Year	Leukemia Type	Dataset	Feature extraction Techinque	ML or DL model	Results
[47]	2022	ALL	C-NMC	Custom CNN feature extraction	ALNett (Cluster-layer CNN)	91.13 % Accuracy, 0.96 F1-score
[48]	2023	ALL(B-cell)	C-NMC, ALL-IDB2	-	VGG16, ResNet50, DenseNet121, EfficientNetB0	C-NMC dataset with high imbalance accuracy:68.61 %
[60]	2024	ALL	C-NMC	AlexNet	OCNN	Accuracy 99.99 %
[50]	2024	ALL	C-NMC	DRDB, GLFEB, CSAB	DDRNet	91.98 % test Accuracy, 0.96 f1-score
[51]	2022	ALL, AML	C-NMC	AlexNet (MSE, HOG, LBP)	SVM	Accuracy: 99.30, Precision: 99.45%, Recall: 99.00%
[52]	2023	ALL	C-NMC	Oversampling, DensNet-201, GRU-BiLSTM	MSVM classifier	Accuracy: 96.29%, f1-score: 96.23%
[53]	2023	AML, ALL	ALLIDB1, ALLIDB2, C-NMC, ASH	ResNet18	ResNet18 + Orthogonal Softmax	ALLIDB1: 99.39%, ALLIDB2: 98.21%, C-NMC: 91.56%, ASH: 97.50%
[54]	2022	B-ALL, T-ALL	ASH dataset	-	AlexNet, LeukNet	Accuracy 94.12 %
[55]	2022	AA, MDS, AML	ASH image bank, private dataset for validation	ResNet-50 CNN	ResNet-50 CNN	**Binary (MDS):** AUC 0.985, Acc 91.4%, Sens 99.2%, External validation Acc 93.5%
[56]	2022	ALL, AML	ALLIDB1, ALLIDB2, ASH	MobileNetV2	SVM	ALLIDB1: 99.39, ALLIDB2: 98.21, ASH: 97.73
[57]	2024	ALL, AML	ALLIDB1, ALLIDB2, ASH	Feature fusion based stacked ensemble with EfficientNetB7 and MobileNetV3, Grad-CAM visualization	EfficientNetB7 and MobileNetV3	Accuracy: 99.3% for multiclass classification
[58]	2025	AML subtypes (M1, M5, M6)	ASH	Inception V3	Inception V3 + GELU for accuracy, ResNet50v2 for computational efficiency	Inception V3 + GELU: 94.02, ResNet50v2: 92.53
[59]	2023	Early pre-B, Pro-B, Pre-B	ALL dataset on Kaggle	EGWO uses K-means clustering for feature selection	Random Forest (Best), KNN, SVM, Naïve Bayes	99.22%
[44]	2021	B-ALL, early pre-B, pre-B, pro-B	ALL dataset on Kaggle	DenseNet-201 after segmentation	Two fully connected NN with four class classification	99.85%
[60]	2024	B-ALL, early pre-B, pre-B, pro-B	ALL dataset on Kaggle	Transfer learning with pre-train ed ResNet-50	ResNet-50 with 5-fold cross validation	Accuracy: 99.38, Precision: 99.39, Recall: 99.38, F1-score: 99.38
[61]	2022	AML: M1/M2	CPTAC-AML (TCIA)	Morphological & radiomics features	Random Forest (Best), BLS, ANN, Naive Bayes, SVM	RF: 99.8%, BLS: 97.3%
[62]	2022	AML, ALL	AML-cytomorphology_LMU (TCIA), PBS-HCB, ALL_IDB2	VGG16-adapted fine-tuned feature extractor	LeuFeatx + XGBoost (Best), SVM, RF, ETC	XGBoost: 92%, SVM: 91.62%
[63]	2021	ALL, AML,	ALL-IDB, AML-cytomorphology_LMU (TCIA)	-	2DCNN	Binary: ALL: 0.97 ± 0.02, AML: 0.93 with early saturation Multiclass (ALL) : F1-score: 0.81 ± 0.09
[64]	2024	AML, Immature WBC into Myeloblasts, Monoblasts, Erythroblasts, Promyelocytes	AML-cytomorphology_LMU (TCIA)	Convolutional Autoencoders, VGG19 and ResNet50	Weighted ensemble of VGG19 and ResNet50	**Binary:** 99.2% accuracy, 99.7% AUC; **Multiclass:** 99.56% accuracy, 99.9% AUC
[65]	2022	AML	AML_Cytomorphology_LMU and	CMYK-Moment Localization, CNN-based feature fusion	RF, SVM, XGBoost	Accuracy: 1st dataset: 97.57(RF), 2nd dataset: 96.41(SVM)
[66]	2023	B-ALL	Blood Cells Cancer (ALL) dataset, Kaggle, (Tehran hospitals, 3242 images)	MobileNetV2	MobileNetV2	Accuracy 100%
[67]	2023	ALL, AML, CML, MM, Lymphoma	Bone marrow microscopic images (China), ALL-IDB1 and SN-AM	Stain domain augmentation	MobileViTv2	Accuracy 94.28 %
[68]	2021	AML, ALL	Private Dataset (EDTA blood smear dataset)	VGG16, ResNet101, DenseNet121	ALNet (VGG16, ResNet101, DenseNet121, SENet154)	Myeloid: Sensitivity 100%, Specificity 92.3%, Precision 93.7% Lymphoid: 89%, 100%, 100%
[69]	2022	ALL, AML	18 public datasets such as ALL-IDB 1 and ALL-IDB2, ASH etc.	Data augmentation	CNN Ensemble (Best)	Multi-level CNN: 94.73%, Ensemble CNN: 94.59%
[70]	2022	AML, ALL	Private Dataset (AIIMS, Patna) + ALL-IDB	LBP, HOG	ResNet50 + SVM (Best), VGG16, DenseNet121	ResNet50 + SVM: 96.67%
[71]	2023	ALL, AML, CLL, CML	Private Dataset (AIIMS, Patna) + ALL-IDB	VGG16	VGG16 + SVM	Accuracy 84%
[72]	2021	ALL	Private dataset (Bone marrow images) (1732) (Shanghai)	-	ResNext	Accuracy 89%
[73]	2023	ALL (B-Cell, T-Cell)	Private dataset (Diagnosis based and symptoms based)	PCA, NMF, Morphological, Texture & Statistical Features	DL Model	Accuracy 92.58%
[74]	2022	APL (AML subtype)	Private Dataset (AIDA2000, NAPOLEON, SAL registry)	-	CNN	APL vs Non-APL AML: 0.85, APL vs Healthy: 0.95
[75]	2021	AML	Private Dataset (Multicenter trials dataset) (Germany)	FRCNN	FRCNN	AML detection: accuracy: 91%, AUC-ROC: 0.97 NPM1 mutation detection: accuracy 86%, AUC-ROC: 92%
[76]	2022	AML, ALL, CML, CLL, MM, MDS	Bone Marrow dataset from Taiwan University hospital	ResNet-101	Cascade R-CNN	Accuracy: 98.9%, Recall: 0.90
[77]	2024	ALL (Early pre-B, Pro-B, Pre-B)	Private dataset (PBS images from Taleqani Hospital, Iran)	HSV based segmentation	ResNet-152 (Best), AlexNet, DenseNet	Accuracy 99.95%

Multipath GAN and augmentation for stain normalization, the MobileViTv2 model that integrates CNN and transformer modules attains 94.28 % accuracy.

[68] uses the EDTA blood smear dataset of 16,450 single-cell images from 731 smears (100 healthy, 191 infected, 148 leukemic), four CNNs (VGG16, ResNet101, DenseNet121, SENet154) are evaluated. VGG16 performs best in a two-module system: the first detects abnormal promyelocytes and distinguishes blasts; the second differentiates myeloid from lymphoid cells. In [70] 500 blood smear images of 44 patients (AML, ALL, and normal) are used to train pre-trained DenseNet121 with SVM with 98% accuracy for binary classification and ResNet50 with SVM with 96.67 % accuracy for three-class classification using a heterogeneous dataset with the ALL-IDB dataset.

[71] utilized a data set of 1250 images (250 images per class in CLL, AML, ALL, and AML) is employed with a VGG16 fine-tuned (last three convolutional layers) and an SVM classifier achieving a performance of 84%. Grad-CAM visualization is employed to emphasize image areas having an effect on classification.

An LLCM diagnostic system for leukemia is created utilizing 1732 bone marrow images of 89 childhood leukemia patients in [72]. A two-phase ResNeXt101-based classification is employed: phase one screens out diagnostically irrelevant images, and phase two classifies 19 WBC types based on an ensemble of ResNeXt101_32×8x, ResNeXt50_32×4d, and ResNet50 with 89% accuracy. ALL diagnosis is deduced from lymphoblast counts.

[74] A deep learning system is built to detect Acute Promyelocytic Leukemia (APL), a subtype of AML, from bone marrow smears without molecular testing. It employs Faster R-CNN for segmentation, Xception-based CNN for classification at the cell level, and ENN for classification at the image level, trained on a private dataset of 51 APL, 1048 non-APL AML patients, and 236 healthy donors.

A model of deep learning is trained to identify AML and forecast NPM1 mutation status from bone marrow smear images. With data on 1251 AML patients (386 with NPM1 mutation) and 236 controls, Faster R-CNN is utilized for segmentation and classification. It has 91 % accuracy in detecting AML and 86 % accuracy in predicting NPM1 mutation [75].

[76] DL framework is designed to perform bone marrow nucleated differential cell count (BM NDC) on high-resolution whole-slide images eliminating the need for manual microscopy. The model typically classifies the Bone-Marrow level cell classification into 16 cell types. BM NDC is crucial for diagnosing and monitoring hematolgic disorders like AML, ALL, MDS, CML and MM depending on the values of cell counts. This study involves a three-stage hierarchical deep learning system: BM particle and cellular trail localization, patch-based cell detection and classification, and fast stitching algorithm. The model identifies 16 clinically relevant BM cell types across myeloid, erythroid and lymphoid lineages. The model is trained using ResNet-101 feature extractor and cascade R-CNN classifier on private dataset and achieves 0.90 recall and 98% accuracy.

[77] A deep ensemble model-based web diagnostic system classifies PBS images into malignant and benign. A ResNet-152 is used as the base learner and ensembled using weighted average voting, with 99.95 % accuracy.

## Analysis of methodological approaches and dataset usage for leukemia studies

**Fig. 3:**
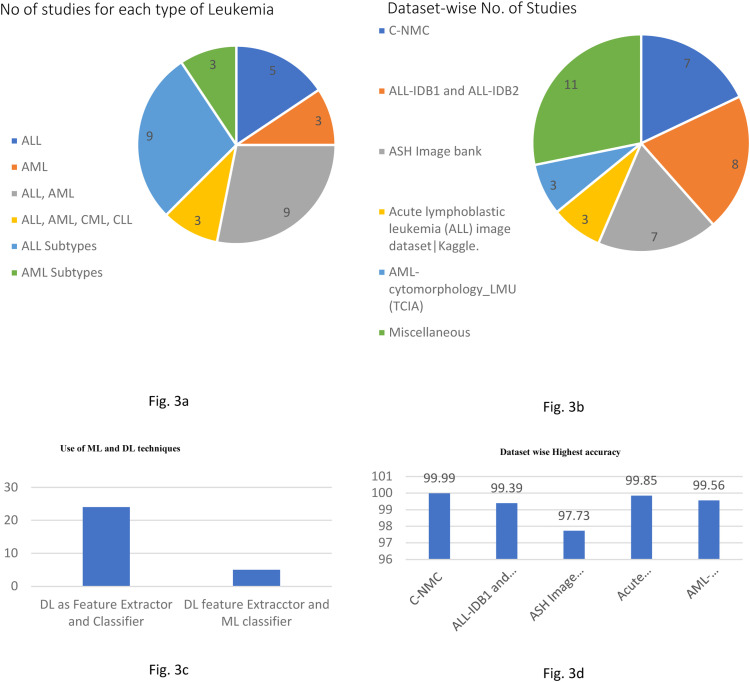
Statistical analysis of Leukemia studies. 3a. Dataset wise Number of Studies 3b. No of Studies for each type of Leukemia 3c. Use of ML and DL techniques 3d. Dataset wise Highest Accuracy.

Fig. 3b shows the dataset-wise number of studies utilized in the literature. The ALL-IDB1 and ALL-IDB2 appeared 8 times, c-NMC and ASH has 7 number of studies each. ALL dataset on Kaggle and AML_Cytomorphology_dataset has 3 numbers of studies each whereas few private datasets and CPTAC-AML dataset comprises 11 studies. The total count doesn’t match the total number of studies as some studies have utilized multiple datasets.

Fig. 3a illustrates the frequency of studies on different types of leukemia. The studies ae categorized as ALL, AML, ALL-AML, ALL-AML, CLL-CML, ALL subtypes and AML subtypes.

The number of studies has used DL for feature extraction as well as classification are 24 whereas there are 5 studies that have utilized DL as feature extractor and ML as a classifier that is shown in Fig. 3c.

It is essential to know the performance achieved by various datasets. Here accuracy has been considered as performance measure as many studies has utilized the same. Fig. 3d shows the highest accuracy achieved by the popular datasets. C—NMC has achieved 99.99%, ALL from Kaggle has 99.85%, AML_Cytomorphology_LMU has achieved 99.56% and ALL-IDB1 and ALL-IDB-2 has 99.39% while ASH has performed with 97.73% accuracy. Even if these studies have achieved the best accuracy it is essential to notice how that systems are developed. Most of the studies have used the GPU enabled systems also many studies have worked on smaller amount of data and they are not tested with real word unknown data (Fig. 3).

## Research gaps

1. **Dataset Limitations**: Most of the studies rely on small, imbalanced or private dataset from a single medical institute dataset that leads to overfitting and poor generalization to real world data. There is no ready dataset available if you have to design a complete hierarchical leukemia classification system. ALL-IDB dataset has only ALL images with subtypes labelled with FAB classification system. C—NMC dataset has only ALL images with subtype labels of B-cell leukemia only. ASH image bank has few images ready to download but they are small in number and also follows the FAB classification system for annotations. AML_Cytomorphology_LMU dataset has the labels with white blood cell morphology only related to AML. CPTAC-AML dataset has only M1 and M2 types (FAB) of AML. Leukemia_attri dataset has all four types of leukemia images but again that lacks the availability of subtype labels.
  So, in summary, there is no dataset available that has the all types and subtypes of leukemia and genomic consideration that is required for WHO standards.
2. **Subtype classification challenges**: Even if public datasets have achieved higher accuracy for binary classification, the model performs poor on subtype and lineage-level classification.
3. **Lacks WHO standards**: WHO revises classification standard every few years and for Leukemia classification recent classification system is updated in 2022 that is more specific to genetic criteria and morphological features but many systems rely on morphological features only. In addition, the dataset annotations follow the FAB classification standards for subtypes labels specially for AML subtypes.
4. **Lack of Explainability and Domain Adaptation**: Most Deep Learning model outperformed on seen datasets. But when it comes to unseen dataset they might perform poor as the images captured can be from different device with different configuration that should be matched with designed system or there should be some provision added to convert the new image to the specific format image that is required by the input for designed deep learning model. The model performance also should be validated clinically and it is not mentioned by any of the studies.
5. **Performance metrics**: Accuracy is considered as the common and widely used performance measure. But when it comes to detection and classification of a life-threatening disease like leukemia, the false negative values should be taken care of and recall should be good enough for the same which is not considered by many of the studies.
6. **Computational complexity**: Many studies have used heavy models like VGGNet, ResNet that requires high end GPU to train and deploy the model which is not adequate every time. Few studies have utilised lightweight models but again the models are trained on small amount of data.

## Future scope

1. Development of comprehensive multimodal datasets: A Multi-modal dataset can be developed that follows WHO 2022 classification standards which focuses both on morphology and the genetic markers considering the complete classification system of leukemia from types to subtype level.
2. Development of hierarchical classification framework: To develop a hierarchical classification framework that can be adopted easily by medical practitioners for real time screening: hierarchical classification system can be developed that will first detect leukemia then it will classify the type of leukemia and then will find out the subtype. To detect the subtype of the leukemia some genetic tests and the flow cytometry with immunophoto typing is performed. Once leukemia is diagnosed this hierarchical classification system would assist medical practitioners to start the treatment early.
3. Adaptation of WHO standards in Dataset Curation and AI models:
4. As genetic markers as well as morphological features are important for classification as per the WHO 2022, they need to be adapted wherever possible.
  Adding Explainability to AI models: Explainability techniques such as GRAD-CAM, SHAP, heatmap can be used to highlight the morphological features that influence the decision which will increase the clinical trust.
5. Clinical Validation and Real-World Deployment with Domain Adaptation: The developed model should undergo cross-dataset validation and should be easily deployable and used. The flexible model can be developed in such a way that input image taken with different equipment and resolution will easily be processed efficiently.

The future of the study has vast amount of work to do as hematological malignancies are not just limited to leukemia but there is lymphoma, multiple myeloma and anemia related disease. If such all in one system is designed it would be easy for the medical practitioners to adopt this system as real-world assistance in screening and diagnosis of blood related disorders.

## Conclusion

This review illustrates the way leukemia diagnosis could be transformed by machine learning and deep learning. Fastermore precise, and less intrusive diagnostic solutions are promised by AI-driven methods that automate feature extraction, classification and therapy recommendations. The ML techniques such as Support Vector Machines and Random Forests provide fundamental insights into data-driven leukemia diagnosis, whereas CNNs and other DL models are excellent at processing vast, unstructured datasets.

Emerging techniques, including ensemble learning and transfer learning, have further enhanced diagnostic capabilities, achieving remarkable accuracies in detecting ALL subtypes. However, significant challenges persist, particularly in dataset availability, model interpretability, scalability, and clinical integration. Addressing these gaps requires collaborative efforts between researchers, clinicians, and policymakers. The future of leukemia diagnostics lies in integrating multimodal data, advancing explainable AI, and developing real-time diagnostic systems that are accessible globally. By overcoming these barriers, AI technologies can significantly improve leukemia patient outcomes, offering personalized, efficient, and reliable healthcare solutions.

## Ethics statements

Not Applicable.

## CRediT author statement

Not Applicable.

## Declaration of interest

The authors declare that they have no known competing financial interests or personal relationships that could have appeared to influence the work reported in this paper.

## References

[1] “Cancer.” Accessed: Jun. 26, 2025 [Online]. Available: https://www.who.int/health-topics/cancer#tab=tab_1.

[2] Wait S. (Sep. 2017). Towards sustainable cancer care: reducing inefficiencies, improving outcomes—a policy report from the All.Can initiative. J. Cancer Policy..

[3] International Agency for Research on Cancer (2020). https://gco.iarc.fr/today/online-analysis-table.

[4] National Cancer Institute (2022). Statistics at a glance: 2022 top 5 most frequent cancers – number of new cases. SEER Cancer Stat. Rev..

[5] B.S. Chhikara and K. Parang, “Chemical biology LETTERS Global Cancer Statistics 2022: the trends projection analysis.” [Online]. Available: https://pubs.thesciencein.org/cbl.

[6] International Agency for Research on Cancer (2020). https://gco.iarc.fr/today/online-analysis-table.

[7] K. Singh, A. Grover, and K. Dhanasekaran, “Unveiling the cancer epidemic in India: a glimpse into GLOBOCAN 2022 and past patterns,” 2025 [Online]. Available: www.thelancet.com. 10.1016/j.lansea.2025.100546 PMC11893321 40070552

[8] Deshantri A.K. (Oct. 2018). Nanomedicines for the treatment of hematological malignancies. J. Control Release.

[9] “Lymphoma - hematology.Org.” Accessed: Sep 30, 2025 [Online]. Available: https://www.hematology.org/education/patients/blood-cancers/lymphoma.

[10] “Myeloma - hematology.Org.” Accessed: Sep 30, 2025 [Online]. Available: https://www.hematology.org/education/patients/blood-cancers/myeloma.

[11] Hassanpour S.H., Dehghani M. (Dec. 2017). Review of cancer from perspective of molecular. J. Cancer Res. Pract..

[12] “The blood and bone marrow | Canadian cancer society.” Accessed: Sep 30, 2025 [Online]. Available: https://cancer.ca/en/cancer-information/what-is-cancer/blood-and-bone-marrow.

[13] Longo D.L. (2017).

[14] “Blood basics - hematology.Org.” Accessed: Aug. 14, 2025 [Online]. Available: https://www.hematology.org/education/patients/blood-basics.

[15] RAY, “Understanding leukemia: A CML survivor’s perspective,” 2019. [Online]. Available: https://www.cmlsurvivor.com/understanding-leukemia. [Accessed: Oct. 3, 2025].

[16] Tigner A., Ibrahim S.A., Murray I.V. (Nov. 2022). Histology, White blood cell. StatPearls.

[17] Aby A.E., Salaji S., Anilkumar K.K., Rajan T. (Sep. 2024). A review on leukemia detection and classification using Artificial Intelligence-based techniques. Comput. Electr. Eng..

[18] “Leukemia - hematology.Org.” Accessed: Jun. 27, 2025 [Online]. Available: https://www.hematology.org/education/patients/blood-cancers/leukemia.

[19] Vardiman J.W., Harris N.L., Brunning R.D. (Oct. 01, 2002).

[20] Vardiman J.W. (Mar. 2010). The World Health Organization (WHO) classification of tumors of the hematopoietic and lymphoid tissues: an overview with emphasis on the myeloid neoplasms. Chem. Biol. Interact..

[21] Arber D.A. (May 19, 2016).

[22] Khoury J.D. (Jul. 01, 2022).

[23] Alaggio R. (Jul. 01, 2022).

[24] Li W. (2022).

[25] Scotti F. (Sep. 2011). Proc. 2011 18th IEEE Int. Conf. Image Process. (ICIP).

[26] Chiaretti S., Zini G., Bassan R. (2014).

[27] G. Abdul-Hamid, “Classification of acute leukemia,” 200 AD [Online]. Available: https://www.intechopen.com [Accessed: Oct. 3, 2025].

[28] Lin Q. (Dec. 2020). Global, regional, and national burden of Chronic myeloid leukemia, 1990–2017: a systematic analysis for the Global burden of disease study 2017. Front. Oncol..

[29] Ferrari B., Peyvandi F. (Nov. 2020). How I treat thrombotic thrombocytopenic purpura in pregnancy. Blood.

[30] Hu Y. (Feb. 2022). Global burden and attributable risk factors of acute lymphoblastic leukemia in 204 countries and territories in 1990–2019: estimation based on Global Burden of Disease Study 2019. Hematol. Oncol..

[31] Yao Y., Lin X., Li F., Jin J., Wang H. (Dec. 2022). The global burden and attributable risk factors of chronic lymphocytic leukemia in 204 countries and territories from 1990 to 2019: analysis based on the global burden of disease study 2019. Biomed. Eng. Online.

[32] Sun L., Yin Y., Guo X., Yu F., Xu J. (2025). Unilateral sudden hearing loss as the first presenting symptom of chronic myeloid leukemia: a case report and literature review. Front. Oncol..

[33] D.A. Howell et al., “Time-to-diagnosis and symptoms of myeloma, lymphomas and leukaemias: a report from the Haematological malignancy research network,” 2013 [Online]. Available: www.hmds.info. 10.1186/2052-1839-13-9 PMC4176985 24238148

[34] A.S. Davis, A.J. Viera, and M.D. Mead, “Leukemia: an overview for primary care,” 2014 [Online]. Available: www.aafp.org/afp. 24784336

[35] Tripathi A.K., Chuda R. (Jan. 2025). StatPearls.

[36] Haas “V.de (Feb. 2019). Initial diagnostic work-up of acute leukemia: ASCO clinical practice guideline endorsement of the college of American pathologists and American society of hematology guideline. J. Oncol. Pr..

[37] Ambayya A., Razali R., Hassan R. (Sep. 2025). Gene expression profiling and pathway analysis in acute myeloid leukaemia-normal karyotype patients. PLoS. One.

[38] Ambayya A. (Sep. 2025). Gene expression profiling and pathway analysis in acute myeloid leukaemia-normal karyotype patients. PLoS. One.

[39] Elsayed B. (2023). Deep learning enhances acute lymphoblastic leukemia diagnosis and classification using bone marrow images. Front. Media SA.

[40] Inaba H., Greaves M., Mullighan C.G. (Jun. 2013). Acute lymphoblastic leukaemia. Lancet.

[41] Duggal R., Gupta A., Gupta R., Wadhwa M., Ahuja C. (Dec. 2016). ACM International Conference Proceeding Series.

[42] Gupta R., Gehlot S., Gupta A. (May 2022). C-NMC: b-lineage acute lymphoblastic leukaemia: a blood cancer dataset. Med. Eng. Phys..

[43] SN-AM, “The cancer imaging archive (TCIA),” 2025 [Online]. Available: https://www.cancerimagingarchive.net/collection/sn-am/ [Accessed: Sep 25, 2025].

[44] Ghaderzadeh M., Aria M., Hosseini A., Asadi F., Bashash D., Abolghasemi H. (Aug. 2022). A fast and efficient CNN model for B-ALL diagnosis and its subtypes classification using peripheral blood smear images. Int. J. Intell. Syst..

[45] “AML-CYTOMORPHOLOGY_LMU - the cancer imaging archive (TCIA).” Accessed: Sep. 27, 2025 [Online]. Available: https://www.cancerimagingarchive.net/collection/aml-cytomorphology_lmu/.

[46] “CPTAC-AML - the cancer imaging archive (TCIA).” Accessed: Sep 20, 2025 [Online]. Available: https://www.cancerimagingarchive.net/collection/cptac-aml/.

[47] Jawahar M., H S., L J.A., Gandomi A.H. (Sep. 2022). ALNett: a cluster layer deep convolutional neural network for acute lymphoblastic leukemia classification. Comput. Biol. Med..

[48] Depto D.S., Rizvee M.M., Rahman A., Zunair H., Rahman M.S., Mahdy M.R.C. (Jan. 2023). Quantifying imbalanced classification methods for leukemia detection. Comput. Biol. Med..

[49] Talaat F.M., Gamel S.A. (Jan. 2024). Machine learning in detection and classification of leukemia using C-NMC_Leukemia. Multimed. Tools. Appl..

[50] Jawahar M., Anbarasi L.J., Narayanan S., Gandomi A.H. (Dec. 2024). An attention-based deep learning for acute lymphoblastic leukemia classification. Sci. Rep..

[51] Shawly T., Alsheikhy A.A. (2022). Biomedical diagnosis of leukemia using a deep learner classifier. Comput. Intell. Neurosci..

[52] Mohammed K.K., Hassanien A.E., Afify H.M. (Aug. 2023). Refinement of ensemble strategy for acute lymphoblastic leukemia microscopic images using hybrid CNN-GRU-BiLSTM and MSVM classifier. Neural Comput. Appl..

[53] Das P.K., Sahoo B., Meher S. (May 2023). An efficient detection and classification of acute leukemia using transfer learning and orthogonal softmax layer-based model. IEEE/ACM. Trans. Comput. Biol. Bioinform..

[54] Anilkumar K.K., Manoj V.J., Sagi T.M. (Oct. 2022). Automated detection of B cell and T cell acute lymphoblastic leukaemia using Deep learning. IRBM.

[55] Wang M., Dong C., Gao Y., Li J., Han M., Wang L. (Apr. 2022). A deep learning model for the automatic recognition of aplastic anemia, myelodysplastic syndromes, and acute myeloid leukemia based on bone marrow smear. Front. Oncol..

[56] Das P.K., Nayak B., Meher S. (Mar. 2022). A lightweight deep learning system for automatic detection of blood cancer. Meas.

[57] Himel M.H.A.M.H., Hasan M.A.M., Suzuki T., Shin J. (2024). Feature fusion based ensemble of deep networks for acute leukemia diagnosis using microscopic smear images. IEEe Access..

[58] Aby A.E., Salaji S., Anilkumar K.K., Rajan T. (Jan. 2025). Classification of acute myeloid leukemia by pre-trained deep neural networks: a comparison with different activation functions. Med. Eng. Phys..

[59] Sallam N.M., Saleh A.I., Ali H.Arafat, Abdelsalam M.M. (Apr. 2023). An efficient EGWO algorithm as feature selection for B-ALL diagnoses and its subtypes classification using peripheral blood smear images. Alex. Eng. J..

[60] Alim M.S. (Dec. 2024). Integrating convolutional neural networks for microscopic image analysis in acute lymphoblastic leukemia classification: a deep learning approach for enhanced diagnostic precision. Syst. Soft Comput..

[61] Liu K., Hu J. (Aug. 2022). Classification of acute myeloid leukemia M1 and M2 subtypes using machine learning. Comput. Biol. Med..

[62] Rastogi P., Khanna K., Singh V. (Mar. 2022). LeuFeatx: deep learning–based feature extractor for the diagnosis of acute leukemia from microscopic images of peripheral blood smear. Comput. Biol. Med..

[63] Schouten J.P.E., Matek C., Jacobs L.F.P., Buck M.C., Bošnački D., Marr C. (Dec. 2021). Tens of images can suffice to train neural networks for malignant leukocyte detection. Sci. Rep..

[64] Elhassan T. (Oct. 2024). CAE-ResVGG FusionNet: a feature extraction framework integrating convolutional autoencoders and transfer learning for immature white blood cells in acute myeloid leukemia. Heliyon.

[65] Elhassan T.A.M., Rahim M.S.M., Swee T.T., Hashim S.Z.M., Aljurf M. (2022). Feature extraction of white blood cells using CMYK-moment localization and deep learning in acute myeloid leukemia blood smear microscopic images. IEEe Access..

[66] Hosseini A. (Jan. 2023). A mobile application based on efficient lightweight CNN model for classification of B-ALL cancer from non-cancerous cells: a design and implementation study. Inf. Med. Unlock..

[67] Yang G., Qin Z., Mu J., Mao H., Mao H., Han M. (Jul. 2023). Efficient diagnosis of hematologic malignancies using bone marrow microscopic images: a method based on MultiPathGAN and MobileViTv2. Comput. Methods Programs Biomed..

[68] Boldú L., Merino A., Acevedo A., Molina A., Rodellar J. (Apr. 2021). A deep learning model (ALNet) for the diagnosis of acute leukaemia lineage using peripheral blood cell images. Comput. Methods Programs Biomed..

[69] Claro M.L. (Sep. 2022). Assessing the impact of data augmentation and a combination of CNNs on leukemia classification. Inf. Sci..

[70] Abhishek A., Jha R.K., Sinha R., Jha K. (Feb. 2022). Automated classification of acute leukemia on a heterogeneous dataset using machine learning and deep learning techniques. Biomed. Signal. Process. Control.

[71] Abhishek A., Jha R.K., Sinha R., Jha K. (May 2023). Automated detection and classification of leukemia on a subject-independent test dataset using deep transfer learning supported by Grad-CAM visualization. Biomed. Signal. Process. Control.

[72] Zhou M. (Jun. 2021). Development and evaluation of a leukemia diagnosis system using deep learning in real clinical scenarios. Front. Pediatr..

[73] Jiwani N., Gupta K., Pau G., Alibakhshikenari M. (2023). Pattern recognition of acute lymphoblastic leukemia (ALL) using computational deep learning. IEEe Access..

[74] Eckardt J.N. (Dec. 2022). Deep learning identifies acute promyelocytic leukemia in bone marrow smears. BMC. Cancer.

[75] Eckardt J.N. (Jan. 2022). Deep learning detects acute myeloid leukemia and predicts NPM1 mutation status from bone marrow smears. Leukemia.

[76] Wang C.W., Huang S.C., Lee Y.C., Shen Y.J., Meng S.I., Gaol J.L. (Jan. 2022). Deep learning for bone marrow cell detection and classification on whole-slide images. Med. Image Anal..

[77] Perveen S., Alourani A., Shahbaz M., Ashraf M.U., Hamid I. (2024). A framework for early detection of acute lymphoblastic leukemia and its subtypes from peripheral blood smear images using deep ensemble learning technique. IEEe Access..

